# Epigenetic reprogramming in the mammalian germline

**DOI:** 10.18632/oncotarget.6206

**Published:** 2015-10-21

**Authors:** Fei-Man Hsu, Amander Clark, Pao-Yang Chen

**Affiliations:** Institute of Plant and Microbial Biology, Academia Sinica, Taipei, Taiwan

**Keywords:** Chromosome Section, germline, development, DNA demethylation, methylome

The role of the germline is to transmit genetic and epigenetic information from one generation to the next. Primordial germ cells (PGCs) are the pioneering germ cell population in mammals and exist for a relatively short period of time during gestation. PGCs are induced from the epiblast by bone morphogenetic protein 4 (BMP4) signaling. These definitive PGCs then migrate from their site of origin to the developing gonad during gestation. After colonization and sex-determination, they start to differentiate into male and female germ cells, which then become gametes upon spermatogenic and oogenic instructions from the niche. Since PGCs are the only embryonic cell type that is capable of differentiating into gametes, monitoring the epigenome and transcriptome of PGCs could provide important insights into the cell and molecular foundation of germline development and healthy gametes. Even though genetic information from the parental gametes is mostly maintained after fertilization and creation of the diploid embryo, the epigenome is reprogrammed with one of the major events involving the genome-wide reprogramming of DNA demethylation. A second reprogramming event in PGCs is hypothesized to remove the memory of embryo development in the epiblast. Understanding the dynamics of epigenome reprogramming in PGCs could therefore help to detect any environmental effects potentially associating with infertility or abnormal child health.

The dynamics of DNA methylation reprogramming in mouse PGCs was first established by Seisenberger *et al* in 2012 [1]. They collected germ cells across key stages of development including PGCs from embryonic day (E) 9.5 to E13.5, E16.5 germ cells, and E6.5 epiblasts. The CpG methylation level in E6.5 epiblasts was 71%, close to the 74% in embryonic stem cell (ESCs), and droped globally during PGC expansion and migration. The CpG methylation reached the lowest level at E13.5 with an average of 14% in male and 7% in female PGCs. They found that imprinting loci, CpG islands on the X chromosome and germline-specific genes are demethylated late upon entry of PGCs into the gonads between E11.5-E13.5.

There is a very limited relationship between loss of DNA methylation and gain in gene expression, including expression of transposons. In a second comprehensive study Kobayashi *et al* used a post-bisulfite adaptor tagging (PBAT) method with scaled-down amounts of DNA from mouse germ cells at E10.5 to E16.5 [2]. This group observed a similar pattern of global DNA demethylation and *de novo* methylation during PGC development. Specifically, in mouse, after E16.5 only male PGCs have increased amounts of CpG methylation, with non-CpG methylation found around transcription start sites. In contrast, promoter regions are depleted of *de novo* methylation.

The development of human PGCs starts around week 2 after fertilization being specified at the onset of gastrulation. The definitive PGCs migrate from the yolk sac wall to the developing gonads during weeks 3-5, and undergo sex differentiation after week 9. In 2015, three groups published in *Cell* the transcriptional and epigenetic dynamics of human PGC development [3-5] from 4- to 19-week-old human embryos. Compared to human somatic cells, human ESCs and inner cell mass, Gkountela *et al* showed that global DNA demethylation in PGCs occurs before 7 weeks of development. This global loss of DNA methylation is maintained until at least week 16 in females. In contrast the initial wave of re-methylation in the male germline can be seen at 19 weeks of development. The DNA demethylation dynamics during week 5 to 16 parallels that reported in the mouse E10.5 to E13.5. This epigenetic reprogramming is not correlated with transcriptional regulation in both species. In addition, Gkountela *et al* discovered 585 persistently methylated regions that in some cases exhibit increased methylation during reprogramming. These genes are not transcriptionally repressed and may be critical for diverse mechanisms.

Between human and mouse PGCs, some differences are observed: first, mouse PGCs show a persistent enrichment of the repressive chromatin mark H3K27me3 and global loss of H3K9me2, while human PGCs after 9 weeks are depleted of both. Secondly, mouse PGCs undergo imprint erasure after the entry to the gonads whereas this occurs before colonization in humans. Finally, X reactivation takes place with different timing in mouse and human PGCs. Gkountela *et al* also found that *XIST* might be non-silencing in the human germline since *XIST* non-coding RNA is expressed in both male and female germline cells at all stages. These findings indicate that mice and humans exhibit differences in germline development which necessitates studying the formation of this lineage in both species. Since PGCs are unique cells that differentiate into gametes and are critical for the continuation of the species, scientists are eager to create this progenitor cell type in the lab. Using spontaneous differentiation, we have generated PGCs *in vitro* that are equivalent to the pre-gonadal stage [6]. Using directed differentiation Irie *et al.* differentiated PGC-like cells (PGCLC) at an equivalent stage with high efficiency [7]. The resources of the human and mouse PGC methylome and transcriptome not only revealed the dynamics of epigenetic reprogramming in the germline but will also serve as a reference for scientists to generate PGCs *in vitro*. The generation of PGCs *in vitro* will be a tremendous benefit to human reproductive medicine and the discovery of genes or environmental factors that impact formation of high quality gametes.
